# Status of Surgical Management of Borderline Ovarian Tumors in France: are Recommendations Being Followed? Multicentric French Study by the FRANCOGYN Group

**DOI:** 10.1245/s10434-021-09852-9

**Published:** 2021-04-26

**Authors:** Lise Lecointre, Virginie Bund, Eva Sangnier, Lobna Ouldamer, Sofiane Bendifallah, Martin Koskas, Pierre-Adrien Bolze, Pierre Collinet, Geoffroy Canlorbe, Cyril Touboul, Cyrille Huchon, Charles Coutant, Emilie Faller, Thomas Boisramé, Justine Gantzer, Martin Demarchi, Vincent Lavoué, Chérif Akladios

**Affiliations:** 1 Department of Gynecologic Surgery, Hôpitaux Universitaires de Strasbourg, Strasbourg, France; 2 I-Cube UMR 7357-Laboratoire des Sciences de L’ingénieur, de L’informatique et de L’imagerie, Université de Strasbourg, Strasbourg, France; 3 Institut Hospitalo-Universitaire (IHU), Institute for Minimally Invasive Hybrid Image-Guided Surgery, Université de Strasbourg, Strasbourg, France; 4 Laboratoire d’ImmunoRhumatologie Moléculaire, Institut National de la Santé et de la Recherche Médicale (INSERM) UMR_S 1109, Institut thématique interdisciplinaire (ITI) de Médecine de Précision de Strasbourg, Transplantex NG, Faculté de Médecine, Fédération Hospitalo-Universitaire OMICARE, Fédération de Médecine Translationnelle de Strasbourg (FMTS), Université de Strasbourg, Strasbourg, France; 5 Department of Gynecology, Hôpitaux Universitaires de Reims, Reims, France; 6 Department of Gynecology, Hôpital Universitaire de Tours, Tours, France; 7 Department of Gynaecology and Obstetrics, Hôpital Tenon, AP-HP, Paris, France; 8 Department of Gynecology, Hôpital Bichat, AP-HP Paris, France; 9 Gynecological Surgery Service, CHU Lyon-Sud, Pierre-Bénite, Lyon, France; 10 Department of Gynecological Surgery, Hôpital Jeanne De Flandre, CHRU, Lille, France; 11 Department of Gynecologic and Breast Surgery and Oncology, Hôpital la Pitié Salpétrière, AP-HP, Paris, France; 12 Department of Obstetrics and Gynaecology, Centre Hospitalier Intercommunal, Créteil, France; 13 Department of Gynecology, Centre Hospitalier de Poissy, Poissy, France; 14 Department of Surgical Oncology, Georges-Francois Leclerc Cancer Center, Dijon, France; 15 Department of Medical Oncology, Hôpitaux Universitaires de Strasbourg, Strasbourg, France; 16 Medical Oncology Department, Centre Paul Strauss, Strasbourg, France; 17 Department of Gynecologic Surgery, Hôpital Universitaire de Rennes, Rennes, France

## Abstract

**Background:**

Borderline ovarian tumors (BOTs) are tumors with a favorable prognosis but whose management by consensus is essential to limit the risk of invasive recurrence. This study aimed to conduct an inventory of surgical practices for BOT in France and to evaluate the conformity of the treatment according to the current French guidelines.

**Methods:**

This retrospective, multicenter cohort study included nine referral centers of France between January 2001 and December 2018. It analyzed all patients with serous and mucinous BOT who had undergone surgery. A peritoneal staging in accordance with the recommendations was defined by performance of a peritoneal cytology, an omentectomy, and at least one peritoneal biopsy.

**Results:**

The study included 332 patients. A laparoscopy was performed in 79.5% of the cases. Treatment was conservative in 31.9% of the cases. The recurrence rate was significantly increased after conservative treatment (17.3% vs 3.1%; *p* < 0.001). Peritoneal cytology was performed for 95.5%, omentectomy for 83.1%, and at least one biopsy for 82.2% of the patients. The overall recurrence rate was 7.8%, and the recurrence was invasive in 1.2% of the cases. No link was found between the recurrence rate and the conformity of peritoneal staging. The overall rate of staging noncompliance was 22.9%.

**Conclusion:**

The current standards for BOT management seem to be well applied.

Borderline tumors of the ovary (BOTs), first described by Taylor^1^ in 1929, account for 10% to 20% of ovarian tumors.^2^–^4^ According to the World Health Organization (WHO) definition, a borderline tumor is has no obvious invasion of the stroma and exhibits both mitotic activity and nuclear abnormalities ranging from clearly benign to indisputably malignant tumors of similar cell type.^5^,^6^

The group most affected by BOTs are women younger than 40 years who may not yet have completed their parental project.^7^ Although the survival rate is excellent, estimated to be 100% at 5 years and 92% at 10 years, the estimated recurrence rate is between 9 and 34% depending on the series and the type of surgical management.^7^–^9^

Conservative surgery currently is known and accepted to be a desirable alternative for young women.^9^,^10^ The challenge of surgical management then is to allow conservative treatment associated with complete peritoneal staging. For this, it is necessary that surgical management be consensual to limit the risk of recurrence in an invasive manner, in an optimal manner from the initial stage, and in accordance with the recommendations^11^ updated in 2020 by the French National College of Obstetricians and Gynecologists (CNGOF).^12^ We used the previous recommendations for comparison because these were the recommendations in effect at the time of patient management. The main objective of this study was to conduct an inventory of surgical practices for BOT in France. The secondary objective was to evaluate the conformity of the treatment according to the current French guidelines.

## Materials and Methods

A retrospective, multicenter cohort study was conducted in the following nine referral centers of France constituting the FRANCOGYN study group: Tenon hospitals, Jean Verdier, Créteil, Poissy and La Pitié Salpêtrière in Paris and the Parisian region, as well as the University Hospitals of Lille, Rennes, Tours, and Strasbourg. All the women gave their consent to participate in the study. The research protocol was approved by the Ethics Committee for Research in Obstetrics and Gynecology (CEROG 2016-GYN 1003).

The study analyzed the medical records of all patients with histologically confirmed mucinous and serous BOT subtypes at all stages according to the Federation of Gynecology and Obstetrics (FIGO) classification diagnosed between January 2001 and December 2018. Among these patients, we included those who at the initial assessment had undergone surgery. The study excluded invasive ovarian tumors discovered during definitive anatomo-pathologic examination, tumors of an endometriosis subtype, patients with incomplete medical records, BOT discovered during pregnancy, and patients who did not undergo surgery. The study enrolled 332 patients.

The following clinical and demographic variables were collected: age at diagnosis, body-mass index (BMI), hormonal status, parity and gestity, mode of diagnosis, preoperative assessment performed, and preoperative CA125 level. If CA125 was 35 IU/mL or greater, it was considered positive. Tumor characteristics also were detailed, namely, FIGO stage^13^ and histologic data of tumors. Surgical procedures were performed (approach, extemporaneous examination, peritoneal cytology, procedures performed).

The French recommendation is a reference framework for the management of BOT initially published in 2002 by the team at the Gustave Roussy Institute^14^ and updated in the 2018 recommendations,^15^ which will be our reference in this article.

### Statistical Analysis

Descriptive analysis of all the included patients was performed. Patient characteristics, tumor characteristics, and operative findings were compared using Student’s *t* test for quantitative variables and Fisher’s exact tests for qualitative variables.

All statistical tests were two-sided, and the significance level was set at 0.05. Statistical analyses were performed using SAS Studio (SAS Institute, Cary, NC, USA).

## Results

### *Characteristics of the Cohort (Table **1**)*

**Table 1:** Patient characteristics

	*n* = 332 *n* (%)
Median age at diagnosis : years (range)	48.8 (16–92)
Median BMI: kg/m^2^ (range)	26.9 (15.6–63)
Nulliparity	109 (32.8)
Median gestity (range)	2.1 (0–16)
Median parity (range)	1.6 (0–8)
Menopause	143 (43.1)
*Mode of discovery*	
Fortuitous	148 (44.6)
Pain	120 (36.1)
Increase in abdominal perimeter	38 (11.4)
Annex torsion	12 (3.6)
Pelvic weight	12 (3.6)
Ultrasound	303 (91.3)
Median tumor size: cm (range)	11.8 (1–42)
Bilaterality	63 (19)
CT scan	130 (39.2)
Presurgery	90 (27.1)
Pelvic MRI	209 (63)
Presurgery	149 (44.9)
Suspicion of BOT	115 (55)
CA-125	274 (82.5)
Median average rate: UI/mL (range)	537 (3–28513)
Rate ≥ 35	147 (53.6)
*Anatomopathology*	
Serous tumor	175 (52.7)
Mucinous tumor	144 (43.4)
Intestinal	78 (/144) (54.2)
Endocervical (seromucinous)	28 (/144) (19.4)
Peritoneal implants	54 (16.3)
Invasive	7 (2.1)
*Procedure at first surgery*	
Laparoscopy	264 (79.8)
With laparoconversion	61 (18.4)
Laparotomy	67 (20.2)

*BMI* body-mass index, *CT* computed tomography, *MRI* magnetic resonance imaging, *BOT* borderline ovarian tumor

The study enrolled 332 patients from the FRANCOGYN database of BOT with an average age at diagnosis of 48.8 years (range, 16–92 years). Of these patients, 43% were postmenopausal (*n* = 143). The rate of nulliparity was 32.8% (*n* = 109). An adnexal mass was discovered incidentally in 44.6% of the cases (*n* = 148), and in the context of pelvic pain in 36.1% of the cases (*n* = 120). The tumor was serous in 52.7% of the cases (*n* = 175) and mucinous in 43.4% of the cases (*n* = 144).

### *Preoperative Assessment (Table **1**)*

An ultrasound was performed in 91.3% of the cases (*n* = 303). The average tumor size was 11.8 cm (range, 1–42 cm), and in 19% of the cases the tumor was bilateral (*n* = 63). Pelvic magnetic resonance imaging (MRI) was performed in 63% of the cases (*n* = 209) and suspected BOT in 55% of the cases (*n* = 115). An abdominopelvic computed tomography (CT) scan was requested in 39.2% of the cases (*n* = 130). The mean CA-125 was 537.5 U/ml (range, 3–28513 U/ml).

### *Track First (Table **2**)*

**Table 2:** Comparison of surgical approaches: laparoscopy vs laparotomy

	Laparoscopy *n* (%)	Lapartomy or laparoconversion *n* (%)	*p* Value^a^
No. of patients	203	128	
Median average age: years (range)	47.2 (16–87)	51.5 (17–92)	0.03
Median BMI: (kg/m^2^ (range)	25.9 (15.6–63)	28.5 (18.3–52)	0.009
Menopause	75 (36.9)	66 (51.6)	0.01
Median tumor size: cm (range)	8.7 (1–30)	16.7 (2–42)	< 0.005
Bilaterality	44 (21.7)	20 (15.6)	0.20
*Presumptive FIGO stage*			
FIGO 1	189 (93.1)	116 (90.6)	0.41
FIGO ≥ 2	5 (2.4)	3 (2.3)	1
*Final FIGO stage*			
FIGO 1	163 (80.3)	108 (84.4)	0.38
FIGO ≥ 2	37 (18.2)	18 (14.1)	0.36
Presence of implants	38 (18.7)	17 (13.3)	0.23
Intraoperative rupture	36 (17.6)	14 (10.9)	0.12
Nonconforming staging	52 (25.6)	24 (18.8)	0.18
Recurrence	16 (7.9)	11 (8.6)	0.84

*BMI* body-mass index, *FIGO* international federation of gynecology and obstetrics

^a^*p* < 0.05 was considered significant

Laparoscopy was performed in 79.5% of the cases (*n* = 265), and 23% of these cases were converted to laparotomy (*n* = 61). A first laparotomy was performed in 20.2% of the cases (*n* = 67). Tumor size was significantly greater in the laparotomy and laparoconversion group than in the laparoscopy group (16.7 vs 8.7 cm; *p* < 0.005). The FIGO stages of the tumors and the number of implants found did not differ significantly between the two approaches. The laparoscopy patients had higher rates of intraoperative ruptures (17.6% vs 10.9%; *p* = 0.12) and staging noncompliance (25.6% vs 18.8%; *p* = 0.18), but the differences were not significant.

### Conservative and Nonconservative Treatment (Tables 3 and 4)

Conservative treatment was administered in 31.9% of the cases (*n* = 106). The rate of postmenopausal patients was significantly higher with radical treatment (139 vs 5; *p* < 0.001) (Table 3). Regardless of hormonal status, conservative treatment was more common among the women younger than 40 years than among the women 41–50 years of age (87.7% vs 60%; *p* < 0.001) (Table 4). Tumor size was significantly smaller with conservative treatment than with radical treatment (10.5 vs 12.4 cm; *p* = 0.03).

**Table 3:** Comparison of tumor resection types: conservative vs nonconservative treatment

	Conservative treatment *n* (%)	No conservative treatment *n* (%)	*p* Value^a^
No. of patients	106	226	
Median average age: years (range)	32.1 (16–92)	56 (18–87)	1.69
Median BMI: kg/m^2^ (range)	26.2 (18.3–45.2)	27.1 (15.6–63)	0.3
Menopause	5 (4.7)	139 (61.5)	<0.001
Median tumor size: cm (range)	10.5 (1–30)	12.4 (2–42)	0.03
Laparoscopy without laparoscopie	73 (68.9)	140 (61.9)	0.27
Bilaterality	13 (11.8)	53 (23.5)	0.01
*Presumptive FIGO stage*			
FIGO 1	101 (95.3)	218 (96.5)	0.56
FIGO ≥ 2	2 (1.8)	6 (2.7)	1
*Final FIGO stage*			
FIGO 1	83 (78.3)	203 (89.8)	0.006
FIGO ≥ 2	21 (19.8)	34 (15)	0.27
Presence of implants	22 (20)	33 (14.6)	0.20
Recurrence	19 (17.3)	7 (3.1)	< 0.001
Deaths related to BOT	0	1 (0.4)	1
Nonconforming staging	24 (22.6)	52 (23)	1

*BMI* body-mass index, *FIGO* international federation of gynecology and obstetrics, *BOT* borderline ovarian tumor

^a^*p* < 0.05 was considered significant

**Table 4:** Management of patients younger than 50 years

Age (years)	< 40 *n* (%)	41–50 *n* (%)	*p* Value^a^
No. of patients	114	50	
Hysterectomy	12 (10.5)	44 (88)	< 0.001
Cystectomy	8 (7)	0	0.12
Conservative treatment	100 (87.7)	30 (60)	0.0001
First laparoscopy	100 (87.7)	36 (72)	0.02

^a^*p* < 0.05 was considered significant

### Peritoneal Staging

With regard to peritoneal staging, peritoneal cytology was performed in 95.5% of the cases (*n* = 317), infracolic or infragastric omentectomy in 83.1% of the cases (*n* = 276), and biopsies (at least 1) in 82.2% of the cases (*n* = 273). The average number of biopsies performed was 1.95 (range, 0–6) at the initial surgery and 3.2 (range, 0–6) at restaging. The biopsies were located in the parieto-colic gutters in 40.4% of the cases. In 55.1% of the cases (*n* = 183), a hysterectomy was performed. Adnexectomy was performed for 96.5% of the mucinous tumors (*n* = 139/144). Among the 78 intestinal subtypes (*n* = 78), 85.9% received an appendectomy (*n* = 67).

### Early and Advanced Stages

An omentectomy was performed significantly more often for advanced-stage disease (FIGO > 1) than for the early-stage disease (FIGO 1) (96.3% vs 82.1%; *p* = 0.007). The rate of subgastric omentectomy was higher for the patients with advanced stages of disease, but this difference was not significant (14.8% vs 7.3%; *p* = 0.10). Biopsies were performed for 81.1% of the patients with early-stage disease and 94.4% of the patients with advanced-stage disease (*p* = 0.016). Hysterectomy was performed for 56.8% of the patients with early-stage disease and 46.3% of the patients with late-stage disease (155 vs 25; *p* = 0.18). No lymph node invasion was found in our study. The rate of staging noncompliance was significantly higher for early-stage disease than for advanced-stage disease (25.6% vs 13%; *p* = 0.05) (Table 5).

**Table 5:** Management of early stages (FIGO 1) compared with advanced stages (FIGO ≥ 2)

	Stage FIGO 1	Stage FIGO ≥2	*p* Value*
No. of patients	273	54	
First laparoscopy	210 (76.9)	47 (87)	0.12
First laparotomy	58 (21.2)	8 (14.8)	0.35
Peritoneal cytology	264 (96.7)	53 (98.1)	1
Omentectomy	224 (82.1)	52 (96.3)	0.007
Infra colic	227 (83.2)	48 (88.9)	0.41
Infra-gastrics	20 (7.3)	8 (14.8)	0.10
Biopsy ≥ 1	222 (81.3)	51 (94.4)	0.016
Hysterectomy	155 (56.8)	25 (46.3)	0.18
Pelvic or lumbo-aortic lymphadenectomy	5 (1.8)	3 (5.6)	0.13
Peritoneal restadification	132 (48.4)	31 (57.4)	0.24
Nonconforming staging	69 (25.6)	7 (13)	0.05
Recurrence	12 (4.4)	14 (25.9)	< 0.001

FIGO, International Federation of Gynecology and Obstetrics

^a^*p* < 0.05 was considered significant.

### Recurrences

The mean follow-up time for recurrence was 63.4 months (range, 7–264 months). The overall recurrence rate was 7.8% (*n* = 27), and the recurrence was invasive in 1.2% of the cases (*n* = 4). The mean time to recidivism was 45.8 months (1–264). The survival rate among recurrences was 100% (*n* = 27) (Table 6). The mean age at recurrence was 34.6 years (range, 18–68 years), and 63% of the patients were nulliparous. Histologically, 66.7% of the patients (*n* = 18) were serous, and 33.3% (*n* = 9) were mucinous. Implants were invasive in 11.1% of the cases. In 44.4% of the cases, the tumor was FIGO stage 1 (*n* = 12), and in 51.9% of the cases, it was stage 2 or higher (*n* = 14). Recurrence surgery was required in 85.2% of the cases (*n* = 23). The rate of second recurrence was 1.8% (*n* = 6) (Table 6). We did not find a statistically significant relationship between the recurrence rate and compliance with peritoneal staging (*p* = 0.35). Similarly, we found no significant difference between the laparoscopic and laparotomy in terms of recurrence (Table 2). On the other hand, the recurrence rate was significantly higher with conservative treatment than with radical treatment (17.3% vs 3.1%; *p* < 0.001) (Table 3).

**Table 6:** Characteristics of recurrence

	Recurrence*n* (%)
No. of patients	27
Median average age: years (range)	34.6 (18–68)
Median BMI:^:^ kg/m^2^ (range)	27.5 (18.6–45.2)
Nulliparity	17 (63)
Menopause	5 (18.5)
*CA-125*	
Median average rate: UI/mL (range)	1453 (15–21,624)
Rate ≥ 35	17 (63)
*Anatomopathology*	
Serous tumor	18 (66.7)
Mucinous tumor	9 (33.3)
Peritoneal implants	14 (51.9)
Peritoneal implants invasive	3 (11.1)
*Stage FIGO*	
FIGO 1	12 (44.4)
FIGO ≥ 2	14 (51.9)
Median average recurrence time: months (range)	45.8 (1–264)
Initial conservative treatment	19 (70.4)
History of chemotherapy	6 (1.8)
Recurrence surgery	23 (85.2)

*BMI* body-mass index, *FIGO* international federation of gynecology and obstetrics

### Compliance with Current Recommendations

An extemporaneous examination of fresh tissue was performed at the time of the first surgery in 44% of the cases (*n* = 146). In 2.1% of these cases, peritoneal cytology was not performed (*n* = 3), and in 34.9%, omentectomy was omitted (*n* = 51). In 27.4% of the cases, no biopsy was performed (*n* = 40). In all the surgeries, 256 patients were fully staged, with 177 patients fully staged during the first surgery, and 79 patients fully staged during the second surgery. The overall rate of staging “not in compliance” with recommendations was 22.9% (*n* = 76).

## Discussion

The group most affected by BOTs are young women who have not yet completed their parental project. Currently, conservative treatment of BOT with the goal of preserving fertility is an acceptable alternative to radical surgery, even in the case of advanced BOT with noninvasive implants.^8^,^10^

In our study, 31.9% of the patients had conservative treatment. The vast majority of these patients (87.7%) were younger than 40 years (*p* = 0.0001). This result is in line with current recommendations. French guidelines insist that the laparoscopic route should be preferred, with protection of the parts during extraction and avoidance of tumor rupture,^15^ as stated in the new recommendations published in 2020.^12^

In our population, the laparoscopy was widely preferred (79.5%), both for initial surgery and for restaging surgery, which is in line with current French recommendations. The use of the laparoscopic approach in the management of BOT is debated in the literature. The main limiting factor of this approach is the tumor volume and the rate of intraoperative tumor rupture. The retrospective multicenter study by Poncelet et al.^16^ showed a significantly increased risk of recurrence in the event of intraoperative tumor rupture. In our study, the risk of intraoperative tumor rupture was not significantly increased in laparoscopy, but the tumors in our study were smaller. Similarly, the recurrence rate was not higher when laparoscopy was performed, as in the retrospective multicenter study by Fauvet et al.^17^ involving 358 patients and in a second study of 535 patients.^18^

With regard to survival, Song et al.^19^ did not demonstrate a significant difference in terms of recurrence-free survival (*p* = 0.50) or overall survival (*p* = 0.65) at the end of a median follow-up period of 41.8 months. Laparoscopy can thus constitute an alternative to laparotomy for the treatment of BOTs by performance of the procedure in an equally optimal way to avoid sub-staging and dissemination.

In accordance with the recommendations, an extemporaneous examination was performed in 44% of the cases. However, despite this examination, peritoneal cytology was omitted in 2.1% of the cases, as was omentectomy in 34.9% of the cases and biopsies in 27.4% of the cases.

In the study by Lenhard et al. 20 the need for multiple biopsies to achieve correct staging was underscored by the fact that implants were widely used in the group of patients analyzed, with 42.1% located in the omentum, 31.6% in the peritoneum, and about 10% in the diaphragm and bladder. Current French guidelines do not give a minimum number of biopsies to be performed and do not specify the location of the biopsies, which leads to randomized management from one center to another. However, the data in the literature on the type and number of biopsies to be performed in the case of BOT are insufficient. On the average, the centers represented in our cohort performed two biopsies during the initial surgery and three biopsies during the restorative surgery, mainly in the parietocolic gutters (40.4%).

Peritoneal cytology is a sensitive indicator of ovarian surface and peritoneal involvement and allows detection of subclinical extraovarian disease in a high proportion of patients.^6^ The centers our study performed peritoneal cytology in 95.5% of the cases. This staging procedure therefore appears to have become routine practice and seems to be performed routinely in the centers studied.

In our cohort, an omentectomy was performed in 83.1% of the cases, as recommended. The gold standard for staging is the infracolic omentectomy, with infragastric omentectomy reserved for advanced stages, but we found no link between the performance of an infragastric omentectomy and the FIGO stage of the tumor.

An adnexectomy was performed in 96.5% of mucinous BOTs, which shows the perfect application of this recommendation in our cohort. In addition, for a tumor of an intestinal subtype, a complementary appendectomy is recommended. Indeed, it currently is well established that mucinous ovarian neoplasia associated with peritoneal pseudomyxoma usually represents a dissemination from a primitive appendicular. The ovarian tumor then often resembles an intestinal-type mucinous BOT.^4^,^21^ The incidences of primary appendicular mucinous BOT and metastatic appendicular mucinous BOT malignancy are respectively 0.7% and 0.5%, with no impact on recurrence-free survival (*p* = 0.964) or overall survival (*p* = 0.219).^22^

In our cohort, an appendectomy was performed in 85.9% of the intestinal subtypes, and this was completely in accordance with the recommendations. Hysterectomy was performed in 55.1% of the cases. In most of the cases, this hysterectomy was performed in the early stages (*p* = 0.18), but in women older than 40 years or postmenopausal women (*p* < 0.001). Thus, the practice of hysterectomy appears more related to the hormonal status and age of the patients than to the FIGO stage of the tumor. Indeed, in the 2017 study by Matsuo et al.,^23^ hysterectomy was not associated with survival of FIGO 1 patients regardless of age. In the case of early-stage patients, this practice should be discussed with the patient.

Pelvic and lumboaortic lymphadenectomy was performed in 1.8% of the early-stage cases (*n* = 5). This practice is not in accordance with the recommendations, which call for it to be performed on a case-by-case basis in the case of invasive uterine implants in the context of advanced-stage BOT (2 or 3). Like hysterectomy, the practice of lymphadenectomy is not related to survival in early-stage cases.^23^ We found an overall recurrence rate of 7.8%, with 1.2% of BOTs in the invasive form. This result is much lower than the rates found in the literature, 29% for the noninvasive form and 5.4% for the invasive form.^9^ Our results could be explained by the fact that our patients come from centers that are experts in ovarian pathology.

The recurrence rate appears to be higher after treatment by cystectomy (23.6%; 95% CI, 0.189–0.292%) than after treatment by adnexectomy, even unilateral treatment (9.5%; 95% CI, 0.074–0.123%).^7^ Similarly, Helpman et al.^24^ in their multivariate analysis found an independent association between cystectomy and recurrence (hazard ratio, 2.57; 95% CI, 1.1–6; *p* = 0.029). It should be noted that our patients with recurrence were young, with an average age of 34.6 years. In 51.9% of these patients, the tumor was advanced, and the vast majority of them had received initial conservative treatment (70.4%). This is in line with the data in the literature because it is well established that the prognostic factors for recurrence are age younger than 45 years at diagnosis, minimal residual disease after surgery, conservative treatment, cystectomy, and the FIGO stage of the tumor.^9^–^11^,^14^,^17^,^24^,^25^

The rate of staging noncompliance was significantly increased for the early stages of disease compared with the advanced stages (25.6% vs 13%; *p* = 0.05). This result is related to the fact that omentectomy and biopsies were performed significantly more often in advanced-stage disease than in early-stage disease. We found no significant relationship between the recurrence rate and compliance with peritoneal staging (*p* = 0.35). Similarly, the meta-analysis by Shim et al.^26^ did not show any impact of compliant surgical staging on survival (odds ratio [OR], 0.98; 95% CI, 0.42–2.29; *p* = 0.97) or recurrence (OR, 0.82; 95% CI, 0.54–1.25; *p* = 0.36). In contrast, we reported a significantly higher recurrence rate in the advanced stages of disease (25.9% vs 4.4%; *p* < 0.001).

The benefit of revision surgery for restaging currently is debated in the literature. The French guidelines recommend restaging in the case of serous tumors with a micropapillary component, in the case of simple cystectomy for a mucinous tumor to perform adnexectomy, or in the case of peritoneal exploration that has not been complete and normal.^15^ Similarly, Seong et al.^27^ recommended restaging if the abdominal cavity and peritoneal surfaces have not been thoroughly examined during the first surgery. For Querleu et al.^28^ restorative surgery would be indicated primarily for patients requiring additional surgical treatment, including omentectomy. It also would be indicated for patients who had an inadequate initial examination of the peritoneum or who had a defective surgical technique performed that could result in seeding of the abdominal wall or peritoneal cavity. On the contrary, patients with a well-documented stage 1A tumor who had undergone a complete resection could have this complementary surgery delayed.^28^

In the meta-analysis of Shim et al.,^26^ the authors recommended surgical restaging if histologic features suggested invasive recurrence (invasive peritoneal implant or micropapillary pattern), if the peritoneum was not described as normal, if no systematic exploration was performed during the initial surgery, if gross peritoneal implants were found in the initial surgery, if gross lesions remained after the initial surgery, or if patients were less likely to present for regular follow-up visits. Because BOT restorative surgery does not appear to have an impact on overall recurrence-free survival,^26^ this is a key point in the proper management of BOT included in the new French recommendations published in 2020.^12^ Actually, the performance of tumor marker CA-125 analysis is no longer recommended for the initial diagnosis.^12^

If we had applied this database to the new recommendations published in 2020, we could have surgically avoided eight pelvic and lumboaortic lymphadenectomy for patients in the advanced stages of BOT as well as 155 hysterectomies for patients in the early stages of BOT and 25 patients in advanced stages of BOT. In particular, we could have avoided 70 restagings for complementary surgical procedures such as hysterectomies, lymphadenectomy, or both. Concerning omentectomies, if the new recommendations had been taken into account, the number of omentectomies performed would not have been modified.

## Conclusion

Borderline ovarian tumors are lesions with a particularly favorable prognosis, but management of BOTs by consensus is essential to limit the risk of invasive recurrence in patients who most often are young. This review of French practices has shown that the current guidelines seem to be generally well applied. The management of BOTs could be centralized in expert centers, with surgery performed by experienced gynecologic oncology surgeons to increase the rate of staging compliance after the initial surgery to limit recurrences.
